# A systems pharmacology workflow with experimental validation to assess the potential of anakinra for treatment of focal and segmental glomerulosclerosis

**DOI:** 10.1371/journal.pone.0214332

**Published:** 2019-03-28

**Authors:** Michael Boehm, Eva Nora Bukosza, Nicole Huttary, Rebecca Herzog, Christoph Aufricht, Klaus Kratochwill, Christoph A. Gebeshuber

**Affiliations:** 1 Division of Pediatric Nephrology and Gastroenterology, Department of Pediatrics and Adolescent Medicine, Medical University of Vienna, Vienna, Austria; 2 Institute of Pathophysiology, Semmelweis University Budapest, Budapest, Hungary; 3 Clinical Institute of Pathology, Medical University of Vienna, Vienna, Austria; 4 Christian Doppler Laboratory for Molecular Stress Research in Peritoneal Dialysis, Department of Pediatrics and Adolescent Medicine, Medical University of Vienna, Vienna, Austria; 5 Epsilon 3 Gmbh, Vienna, Austria; University of KwaZulu-Natal, SOUTH AFRICA

## Abstract

Focal and Segmental Glomerulosclerosis (FSGS) is a severe glomerulopathy that frequently leads to end stage renal disease. Only a subset of patients responds to current therapies, making it important to identify alternative therapeutic options. The interleukin (IL)-1 receptor antagonist anakinra is beneficial in several diseases with renal involvement. Here, we evaluated the potential of anakinra for FSGS treatment. Molecular process models obtained from scientific literature data were used to build FSGS pathology and anakinra mechanism of action models by exploiting information on protein interactions. These molecular models were compared by statistical interference analysis and expert based molecular signature matching. Experimental validation was performed in Adriamycin- and lipopolysaccharide (LPS)-induced nephropathy mouse models. Interference analysis (containing 225 protein coding genes and 8 molecular process segments) of the FSGS molecular pathophysiology model with the drug mechanism of action of anakinra identified a statistically significant overlap with 43 shared molecular features that were enriched in pathways relevant in FSGS, such as plasminogen activating cascade, inflammation and apoptosis. Expert adjudication of molecular signature matching, focusing on molecular process segments did not suggest a high therapeutic potential of anakinra in FSGS. In line with this, experimental validation did not result in altered proteinuria or significant changes in expression of the FSGS-relevant genes COL1A1 and NPHS1. In summary, an integrated bioinformatic and experimental workflow showed that FSGS relevant molecular processes can be significantly affected by anakinra beyond the direct drug target IL-1 receptor type 1 (IL1R1) context but might not counteract central pathophysiology processes in FSGS. Anakinra is therefore not suggested for extended preclinical trials.

## Introduction

Focal and Segmental Glomerulosclerosis (FSGS) is a histopathological description for severe sclerotic lesions in the kidney glomeruli and a common cause of nephrotic syndrome characterized by proteinuria, edema, and hyperlipidemia [1]. The etiology, histomorphology and clinical course of FSGS are highly heterogeneous including mutations in podocyte genes, drugs, viruses and hypertension [2]. Some patients respond well to corticosteroids or calcineurin inhibition, but specific therapy is still lacking. FSGS frequently leads to end stage renal disease (ESRD) requiring renal replacement therapy. A proportion of transplanted patients will suffer from disease recurrence in their allograft [3, 4]. Taken together, there is a major need for alternative treatments of FSGS. However, none of the recent clinical trials in this area has resulted in approval of a novel medication [5]. Targeting neglected pathways might be a solution to this challenge [6]. One of these pathways and targets is interleukin (IL)-1, a versatile pro-inflammatory cytokine that also activates T lymphocytes, which are involved in pathomechanisms associated with the nephrotic syndrome [7, 8]. IL-1 has also been found upregulated in podocytes during glomerular injury [9].

Anakinra is an IL-1 receptor antagonist (IL1-RA) that blocks the biologic activity of IL-1 by competing with its binding to the IL-1 receptor type I (IL-1R1), which is expressed in a wide range of cell types. Anakinra has shown to be effective in various inflammatory diseases and is currently licensed to treat rheumatoid arthritis and cryopyrin-associated periodic syndromes (CAPS) [10]http://www.accessdata.fda.gov/drugsatfda_docs/label/2016/103950s5175lbl.pdf) and has recently been commissioned for treatment of additional rare periodic fevers and autoinflammatory diseases [11]. Experimental evidence suggests a role of IL-1 in kidney disease and hypertension [12]. In line with this, anakinra had beneficial effects including reduction of proteinuria in off-label use in patients with Familial Mediterranean fever, rheumatoid arthritis and Muckle-Wells (urticaria-deafness-amyloidosis) syndrome, a subtype of CAPS [13–15]. As the efficacy of anakinra in FSGS has not yet been evaluated [5, 12, 16], we decided to analyze it in a drug repurposing approach by network-based drug-disease target interference at the molecular level [17]. Our approach based on an algorithm using data from scientific literature consolidation integrates information from unbiased data mining and was recently used to test the potential of the immunosuppressive drug tacrolimus in diabetic nephropathy *in silico* [18]. This network-based drug *versus* disease target interference at the molecular level integrates information obtained from unbiased data mining into a disease specific molecular pathophysiology framework.

In this study, a molecular pathophysiology model characterizing the molecular processes associated with FSGS was overlapped with the molecular mechanism of action of anakinra to predict the therapeutic potential for treatment of FSGS by a workflow combining interference analysis, molecular signature matching and experimental validation [18–20].

## Material and methods

### General data sources

Protein coding genes associated with FSGS or anakinra were collected by a literature mining approach based on public domain data. A PubMed search using the respective major medical subject headings (MeSH) term as query string was performed to identify publications of relevance. Protein coding genes explicitly discussed in these publications were extracted from gene2pubmed by filtering based on PubMed ID and taxonomy ID (9606 for human).

### Molecular process models of clinical phenotype and drug mechanism of action

Construction of molecular process models was performed as previously described [21–23]. In brief, two main steps were performed: (a) mapping of a feature signature being either the data set of protein coding genes associated with FSGS or with anakinra on the consolidated protein interaction network, followed by induced subgraph extraction. Nodes with a degree of zero are removed from the subgraph. (b) molecular process identification via utilizing a segmentation algorithm (MCODE with default settings [24]).

### Molecular signature comparison

Matching signature of disease and drug relevant genes reported in scientific literature was used to predict therapeutic potential of anakinra in FSGS. To do so, regulation of a given gene in scientific literature associated with FSGS was compared to that reported for anakinra. Discordant regulation (*i*.*e*. upregulation reported with FSGS and downregulation with anakinra and *vice versa*) was used to assess whether anakinra was expected to reverse the FSGS specific expression and thus to counteract FSGS pathophysiology and ambiguous results were clarified by expert adjudication. Based on the assumption of identification of central aspects of FSGS pathophysiology by topological segmentation, this analysis focused on these molecular clusters, starting with those with the highest number of shared protein coding genes. Eventually contradicting data were resolved in an expert adjudication process.

### Animal experiments

Lipopolysaccharide (LPS) nephropathy was induced by intraperitoneal (i.p.) injection of 12 mg/kg LPS (serotype 0111:B4, Sigma Aldrich, St. Louis, MO, USA) in 0.9% saline into 10 weeks old female Gt(ROSA)^26Sortm4(ACTB-tdTomato,-EGFP)Luo/J^ x hNPHS2Cre mice (kind gift of Prof. Tobias B. Huber) [25]. All animal experiments and handling were in accordance with the Austrian law for protection of animals and approved by the Institutional Committee for Animal Research and Care at the Medical University of Vienna (66.009/0053-II/3b/2014). Animals were housed in Makrolon cages, type II long in enriched environment and fed standard mouse chow and water ad libitum. For anaesthesia 100mg/kg Ketamine and 10mg/kg Xylazine were used. Adriamycin-dependent nephropathy (active compound: doxorubicin) was induced by tail vein injection of 9.8 mg/kg mouse (glomeruli isolation experiment) or 8.85 mg/kg mouse (anakinra therapy experiment) in 10 weeks old female BALB/c [26]. Anakinra (Kineret, Swedish Orphan Biovitrum AB, Solna, Sweden) was i.p. injected daily at a dose of 25 mg/kg mouse in 0.9% saline [27] from day 7 on, Analysis was performed 35 days post induction (glomeruli isolation) or 18 days post induction (urinary collection in anakinra therapy experiment). Control mice were injected with 0.9% saline. At least 6 mice were analyzed per group. Mice were monitored daily for signs of pain, altered movement or reduced food uptake and sacrificed by cervical dislocation.

### Cell isolation, RNA isolation and qPCR

Glomeruli and podocytes were isolated as described before from 3 animals/group [25]. RNA was isolated with the Qiagen RNeasy kit according to manufacturer’s instructions. qPCR for IL1B, COL1A1, NPHS1 and Cyclophilin B, as housekeeping gene for normalization (all primers from Qiagen, Hilden, Germany) were performed according to the manufacturer’s protocol on a CFX96 Real Time System and a C1000 Thermal Cycler (Bio-Rad, Hercules, CA, USA).

### Urinary albumin and creatinine determination

Urinary albumin levels of spot urine were assessed by ELISA (E90-134, Bethyl Laboratories, Montgomery, TX, USA). Creatinine levels were measured with the Creatinine Assay Kit (STA-378, Cell Biolabs, San Diego, CA, USA) according to the manufacturer’s protocols. Urine albumin-to-creatinine ratio (UACR) was calculated (urine albumin (mg/dl) / urine creatinine (g/dl) = UACR in mg/g ≈ albumin excretion in mg/day) to control for variation in urine concentration.

### Data analysis and statistics

Biological pathway enrichment analysis was performed for the set of protein-coding genes using the PANTHER database (www.panther-db.org) as previously described [28]. For statistical evaluation of molecular model interference, the set of protein-coding genes defined in the clinical phenotype molecular process model of FSGS was contrasted to the set of protein-coding genes defined in the anakinra mechanism of action molecular model with respect to molecular feature overlap as previously described [18, 22]. With the set of protein coding genes as background the feature overlap was tested for over-representation using a Fisher's exact test with a significance level set to 0.05. Multiple tests were adjusted using Benjamini-Hochberg correction.

For experimental data, statistical analyses and graphical representations of the results were performed using Prism 7.04 (GraphPad, La Jolla, CA, USA). Values from different groups were compared using one-way ANOVA with Tukey's honest significant difference test as a post hoc; P values lower than 0.05 were considered significant.

## Results

**Fig 1:**
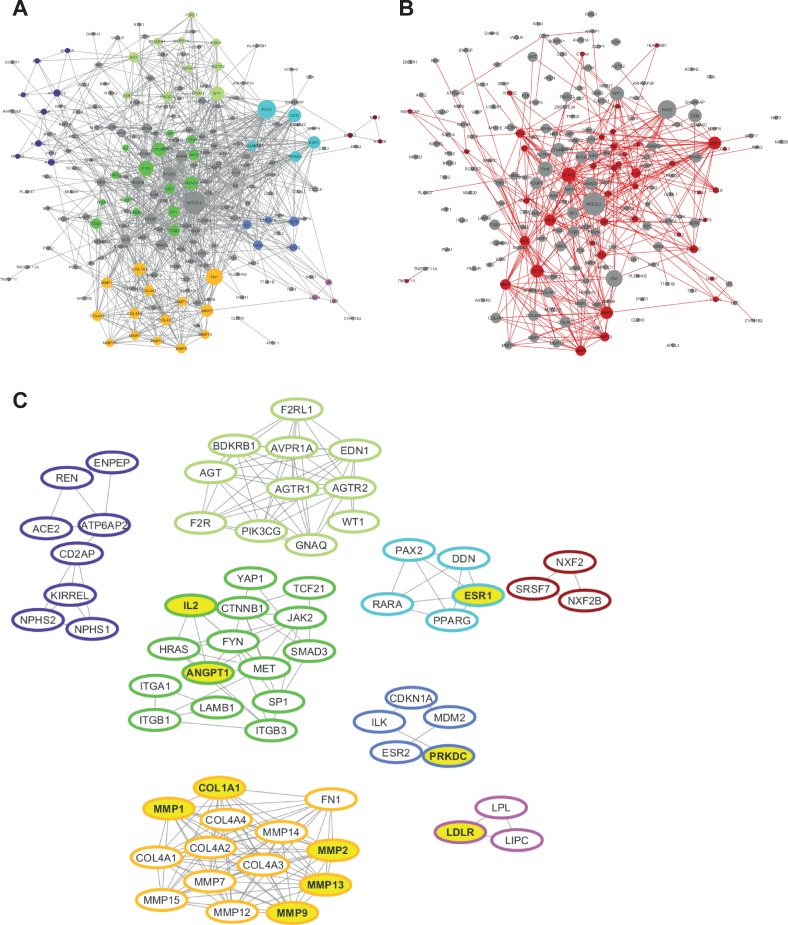
Molecular model interference analysis. (**A**) Molecular process model of FSGS based on an underlying consolidated hybrid protein interaction network. Proteins are represented by nodes, interactions by lines. Eight molecular processes identified by topological segmentation are indicated in individual colors. (**B**) Interference of anakinra mode of action with the model representation of FSGS. Overlapping elements are indicated in red. (**C**) Interference of drug mode of action of anakinra with the eight process segments. Overlapping protein coding genes are indicated in yellow. Interactions between segments (molecular processes) are omitted.

Data mining from scientific literature extracted 225 protein coding genes associated with FSGS that were also part of the consolidated interaction network. 206 protein coding genes were identified as member of the induced subgraph, 19 had no interaction to any other feature of the FSGS data set and were therefore not included in molecular model computation. Following the network segmentation procedure aimed at identifying FSGS molecular process segments defined by topological characteristics of the FSGS-specific subgraph, the constructed molecular process model for FSGS held 64 protein coding genes in 8 process units ranging in size from 3 to 16 protein coding genes (Fig 1A and 1C).

Interfering the FSGS molecular model with anakinra´s mechanism of action gene set on the level of feature overlap (Fig 1B) identified specific protein coding genes of the FSGS molecular model: Of 225 individual protein coding genes of the FSGS model, 43 genes were also part of the drug mechanism of action gene set of anakinra, i.e. predicted to be affected by anakinra (p<0.001). These 43 genes were further subjected to pathway analysis with the PANTHER database. Ten of these overlapping protein coding genes were also part of molecular process segments of FSGS (Fig 1C). Five of these genes (COL1A1, MMP1, MMP2, MMP9, MMP13) were located in the same cluster.

PANTHER pathway analysis (Table 1) revealed *Plasminogen activating cascade* as top enriched of 7 molecular pathways (3 protein coding genes out of the 18 being assigned to this pathway; *p* = 0.0004). Among the list of enriched molecular pathways were signaling cascades linked to inflammation (*Interleukin signaling pathway*, *Inflammation mediated by chemokine and cytokine signaling pathway)* and apoptosis (*Apoptosis signaling pathway*, *FAS signaling pathway*). In addition, *CCKR signaling map* and *Gonadotropin-releasing hormone receptor pathway* were significantly enriched with more than 1 protein coding gene from the constructed FSGS molecular model.

**Table 1:** 

PANTHER Pathways	Reference List	Analyzed List	Expected number			raw P-value	FDR	Genes
				fold Enrichment				
**Plasminogen activating cascade**	18	3	0,04	+	77,93	1,18E-05	3,83E-04	**MMP13, MMP1, MMP9**
**FAS signaling pathway**	34	2	0,07	+	27,51	2,68E-03	4,85E-02	CASP3, CASP8
**Interleukin signaling pathway**	89	4	0,19	+	21,02	4,60E-05	1,25E-03	CXCL8, IL6, STAT3, IL4
**CCKR signaling map**	174	6	0,37	+	16,12	2,20E-06	1,19E-04	CXCL8, BCL2, STAT3, CASP3, NOS1, **MMP9**
**Apoptosis signaling pathway**	120	4	0,26	+	15,59	1,41E-04	3,28E-03	BCL2, TNF, CASP3, CASP8
**Inflammation mediated by chemokine and cytokine signaling pathway**	255	8	0,55	+	14,67	7,61E-08	6,20E-06	CXCL8, CCL3, IL6, VWF, CCL2, IL1B, CCL3L1, STAT3
**Gonadotropin-releasing hormone receptor pathway**	237	4	0,51	+	7,89	1,72E-03	3,50E-02	TGFB1, MIF, STAT3, NOS1
**Unclassified**	18438	21	39,43	-	0,53	4,80E-11	7,83E-09	NTRK2, **LDLR**, TIMP1, AGER, **PRKDC**, IL12B, NOS1, HLADRB1, CASP1, APOE, **ESR1**, FOXN1, **IL2**, MIF, LEP, IL10, TNFSF11, NLRP3, CTLA4, IL12A, ACE

Genes in bold are embedded in molecular process segments.

Matching signature of disease and drug relevant genes, starting in the cluster with the highest number of molecular features shared between the FSGS pathophysiology model and the anakinra mode of action gene data set showed in none of these 5 genes a discordant but in two of them a concordant expression pattern between FSGS and anakinra (MMP2, MMP9).

**Fig 2:**
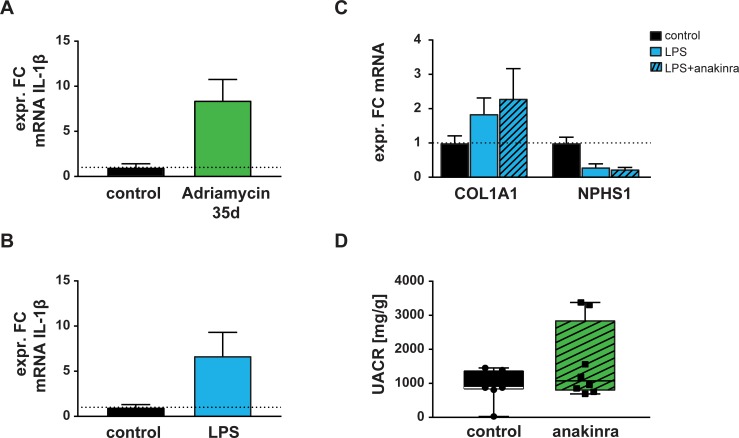
Results of experimental validation of the impact of anakinra in two independent experimental models of FSGS. IL-1β was strongly upregulated in glomeruli of BALB/c mice with Adriamycin-nephropathy (A) and isolated podocytes from mice with LPS-nephropathy (B). (C) Anakinra had no significant impact on the expression of COL1A1 and NPHS1 in isolated podocytes from mice with LPS-nephropathy. (D) Anakinra did not attenuate proteinuria in the Adriamycin-nephropathy model after 35d. FC: fold change; LPS: lipopolysaccharide; UACR: urine albumin-to-creatinine ratio.

Results of experimental validation of the impact of anakinra on experimental models of FSGS are shown in Fig 2. The *in silico* prediction of involvement of IL-1β in the molecular pathophysiology of FSGS was supported as IL-1β was >8-fold upregulated in glomeruli of BALB/c mice with induced Adriamycin-nephropathy for 35d (Fig 2A). In LPS-induced nephropathy, isolated podocytes exhibited a strong increase of IL-1β (Fig 2B), confirming these findings in an unrelated *in vivo* model of FSGS. Treatment with anakinra did not counteract but rather increased the effect of LPS treatment on expression of *COL1A1* encoding for alpha collagen type I, and the podocyte marker nephrin encoded by *NPHS1*, although not significantly (Fig 2C). Finally, in the functional assessment of anakinra effects in the Adriamycin-nephropathy model of FSGS, anakinra did not significantly alter the albumin:creatinine ratio (Fig 2D). Individual values are provided in S1 Table.

## Discussion

Systems pharmacology using computational network analysis has been recently proposed as a novel approach to reposition known drugs for novel indications thereby fostering drug development in areas of yet unmet medical need [29]. In our study, information at the level of protein coding genes associated with disease pathophysiology and with drug mechanism of action was identified by scientific literature consolidation. For bioinformatics analysis we used a human protein coding gene interaction network as framework to integrate information extracted at the level of individual protein coding genes into a molecular functional context [19, 29]. Use of a consolidated network based on INTACT, Reactome, and BioGRID resulted in coverage of more protein coding genes than represented in each individual of these public domain databases [22]. Next, we constructed a specific complex network (= FSGS disease pathophysiology model) by mapping the FSGS-associated protein coding genes (identified by unbiased data mining) on this consolidated protein interaction network, followed by network segmentation. Structuring this network into segments (“molecular processes”) according to topological specifics delineated eight gene clusters, which may reflect particularly relevant aspects from a systems biology perspective [19, 20].

This molecular process model representation of FSGS was used to assess the potential of anakinra as therapeutic option in FSGS [19, 29]. Statistically significant interference of “disease relevant proteins” with “drug target proteins” suggested a possible functional effect. PANTHER pathway analysis was used to generate information about anakinra´s specific mechanism of action in the molecular landscape of the pathophysiology model of FSGS [20]. This analysis predicted high interference in well-established molecular features that are expected within anakinra´s known drug mechanisms, such as *Inflammation mediated by chemokine and cytokine signaling pathway*, in particular the *Interleukin signaling pathway*, and *Apoptosis signaling pathway*, in particular the *FAS signaling pathway* [1, 17]. Other pathways, such as *CCKR signaling map* and *Gonadotropin-releasing hormone receptor pathway* have not been previously associated with FSGS and need to be validated regarding their specific relevance in this context. Interestingly, our analysis also identified FSGS-relevant pathways affected by anakinra beyond those that have been previously described within the direct drug target (IL-1β) context. For example, the strongest overrepresented pathway *Plasminogen activating cascade* suggests high relevance with regards to current FSGS pathophysiology but has not yet been associated with anakinra´s mechanism of action. The soluble urokinase plasminogen activator receptor (suPAR) is intensively discussed as circulating factor causing FSGS [30, 31].

In the next step, we focused on anakinra´s drug mechanism of action applying comparison of molecular signature at the level of shared individual protein coding genes embedded in the molecular process segments–thus reflecting drug effects on central aspects of FSGS molecular pathophysiology [19, 22]. This interference analysis revealed 10 overlapping genes. Five of these genes (COL1A1, MMP1, MMP2, MMP9, MMP13) are associated with extra-cellular matrix (ECM) and were all located in the same cluster suggesting relevant (either positive or negative) impact based on this *in silico* assessment. Qualitative in-depth analysis of scientific literature at the level of expert adjudication was used to allow prediction of the effect of anakinra on FSGS. Matrix metalloproteinases (MMPs) are regulators of ECM turnover and close regulation is essential for ECM homeostasis. MMP2 and MMP9 were found significantly elevated in the urine of patients with steroid-resistant nephrotic syndrome as compared to steroid-sensitive patients [32]. A study analyzing puromycin aminonucleoside (PAN) treated rats also reported the total amount of MMP9 as increased, while the total activity was significantly reduced [33]. Interestingly, levels of MMP9 were reported as concordantly increased with levels of suPAR in inflammatory disorders [34]. Collagen 1 (COL1A1) is a classical marker and promoter of fibrosis/sclerosis and strongly upregulated in human FSGS [35]. Anakinra has been shown to inhibit COL1A1 [36] and to activate several MMPs including MMP1, -2, -9, and -13 [37, 38], but is only one, and not the most crucial, factor in the complex regulation of these genes. Dependent on defined thresholds for definition of suitable drug candidates in systems pharmacology approaches, anakinra would likely not be tested in respective preclinical trials. In this study, however, we decided to validate the effect of anakinra in FSGS to reduce the likelihood of a false negative decision.

FSGS is very heterogeneous and no model covers all major disease nodes relevant in its pathophysiology [16, 39]. To reduce this basic limitation, we chose two unrelated models covering different pathophysiologic aspects of FSGS [26, 40]. Whereas clear upregulation of glomerular IL-1β in both FSGS models suggests a relevant role in FSGS, IL-1β inhibition by anakinra did neither counteract selected molecular processes nor result in functional attenuation of FSGS. In LPS-nephropathy, addition of anakinra appeared to slightly increase the expression of COL1A1, and decrease the podocyte specific marker NPHS1 in isolated podocytes, although both effects did not reach significance. In the long-term model of Adriamycin nephropathy, treatment with anakinra did not reduce proteinuria, the main clinical parameter in FSGS. Thus, in line with our predictions based on *in silico* data two independent mouse models covering different pathophysiologic aspects of FSGS, showed no efficacy of anakinra in FSGS treatment. Our validation assay has several limitations. First, levels of anakinra were neither assessed in blood nor tissue. Second, no positive control arm (such as steroid treatment) was included in the experimental design, and, third, anakinra might still be effective in other genetic mice models due to the multiplicity of etiologic factors in the pathogenesis of FSGS. However, the used dose in the mouse experiments corresponded to a more than 15-fold equivalent of the recommended human daily dose and has previously been reported to be effective [27]. Although improved experimental designs and inclusion of additional experimental models might further increase the experimental rigor to exclude the potential of anakinra as a novel approach to treat human FSGS, we are confident that the combination of two established mouse models in this study should provide sufficient (negative) evidence to focus on other drug candidates for future preclinical development.

In summary, our workflow demonstrated that a molecular network-based systems pharmacology approach might represent a rational strategy for drug repurposing in FSGS. It might thus represent a feasible approach for efficacious development of novel therapies for complex difficult-to-treat diseases such as FSGS due to early exclusion of drug candidates during the *in silico* stage of efficacy analysis [29].

## Supporting information

S1 Table Experimental data, individual values. The table provides values behind the means, standard deviations and other measures reported.
(XLSX)
